# Estimating the accuracy of optic nerve sheath diameter measurement using a pocket-sized, handheld ultrasound on a simulation model

**DOI:** 10.1186/s13089-016-0053-9

**Published:** 2016-11-10

**Authors:** Garrett G. R. J. Johnson, Frederick A. Zeiler, Bertram Unger, Gregory Hansen, Dimitrios Karakitsos, Lawrence M. Gillman

**Affiliations:** 1 Undergraduate Medical Education, University of Manitoba, Winnipeg, MB Canada; 2 Departments of Surgery, University of Manitoba, GF439, 820 Sherbrook Street, Winnipeg, MB R3A 1R9 Canada; 3 Clinician Investigator Program, University of Manitoba, Winnipeg, MB Canada; 4 Department of Internal Medicine, Section of Critical Care, University of Manitoba, Winnipeg, MB Canada; 5 Pediatrics and Child Health, University of Manitoba, Winnipeg, MB Canada; 6 Department of Critical Care, Keck Medical School, USC, Los Angeles, CA USA

**Keywords:** Point-of-care ultrasound, Optic nerve sheath diameter, Ultrasound, Intra-cranial pressure, Handheld ultrasound

## Abstract

**Background:**

Ultrasound measurement of optic nerve sheath diameter (ONSD) appears to be a promising, rapid, non-invasive bedside tool for identification of elevated intra-cranial pressure. With improvements in ultrasound technology, machines are becoming smaller; however, it is unclear if these ultra-portable handheld units have the resolution to make these measurements precisely. In this study, we estimate the accuracy of ONSD measurement in a pocket-sized ultrasound unit.

**Methods:**

Utilizing a locally developed, previously validated model of the eye, ONSD was measured by two expert observers, three times with two machines and on five models with different optic nerve sheath sizes. A pocket ultrasound (Vscan, GE Healthcare) and a standard portable ultrasound (M-Turbo, SonoSite) were used to measure the models. Data was analyzed by Bland–Altman plot and intra-class correlation coefficient (ICC).

**Results:**

The ICC between raters for the SonoSite was 0.878, and for the Vscan was 0.826. The between-machine agreement ICC was 0.752. Bland–Altman agreement analysis between the two ultrasound methods showed an even spread across the range of sheath sizes, and that the Vscan tended to read on average 0.33 mm higher than the SonoSite for each measurement, with a standard deviation of 0.65 mm.

**Conclusions:**

Accurate ONSD measurement may be possible utilizing pocket-sized, handheld ultrasound devices despite their small screen size, lower resolution, and lower probe frequencies. Further study in human subjects is warranted for all newer handheld ultrasound models as they become available on the market.

## Background

Elevated intra-cranial pressure (ICP) is a frequent and grave complication of neurological injury secondary to conditions, such as traumatic brain injury, hydrocephalus, hepatic encephalopathy, and central nervous system infection. Close ICP monitoring is fundamental to the early detection and appropriate management [1]. Direct ventricular or intra-parenchymal monitoring is considered the gold standard for measuring ICP; however, the procedure is invasive, carries the risk of infection and bleeding, and is resource intensive [2, 3]. Other modalities for estimating ICP, such as lumbar puncture, computed tomography, or magnetic resonance imaging, have their own limitations, and can be impractical or dangerous in a variety of patient care settings [4]. A variety of non-invasive techniques utilizing ultrasound have been investigated including transcranial Doppler (TCD) ultrasonography and the pulsatility index calculated from its flow velocity waveform, and transorbital ultrasound measurement of optic nerve sheath diameter (ONSD). Both show promise in the emergency medicine and critical care literature for a variety of adult and pediatric patient populations [5–14]. Ultrasound is readily available, rapid, and non-invasive, making it a potentially excellent tool for monitoring the neurocritically ill.

With improvements in ultrasound technology, machines are becoming ultra-portable allowing for flexible point-of-care examinations, including at the scene of injury, in austere environments and during transport. Recent literature suggests that ONSD measurements by ultrasound show low inter- and intra-observer variability [6, 8, 9, 15, 16]. However, to diagnose increased ICP, ONSD measurements require precise measurement of a 3–6 mm structure to the nearest 0.1 mm accuracy [8, 17] and it is unclear if these ultra-portable handheld units have the resolution to make these precise measurements.

In this study, we estimate the accuracy of ONSD measurement in a pocket-sized ultrasound unit (Vscan, GE Healthcare) utilizing a locally developed model of the eye and optic nerve sheath, and compare it to previously validated portable unit (M-Turbo, SonoSite) as a reference standard.

## Methods

### Model

All measurements were made using our previously developed and validated optic nerve sheath models [16, 18] with varying sheath diameters of known size (simulated from 3-D printed discs), and conducted by expert point-of-care ultrasonographers (LG and GH).

### Ultrasound technique

**Fig. 1:**
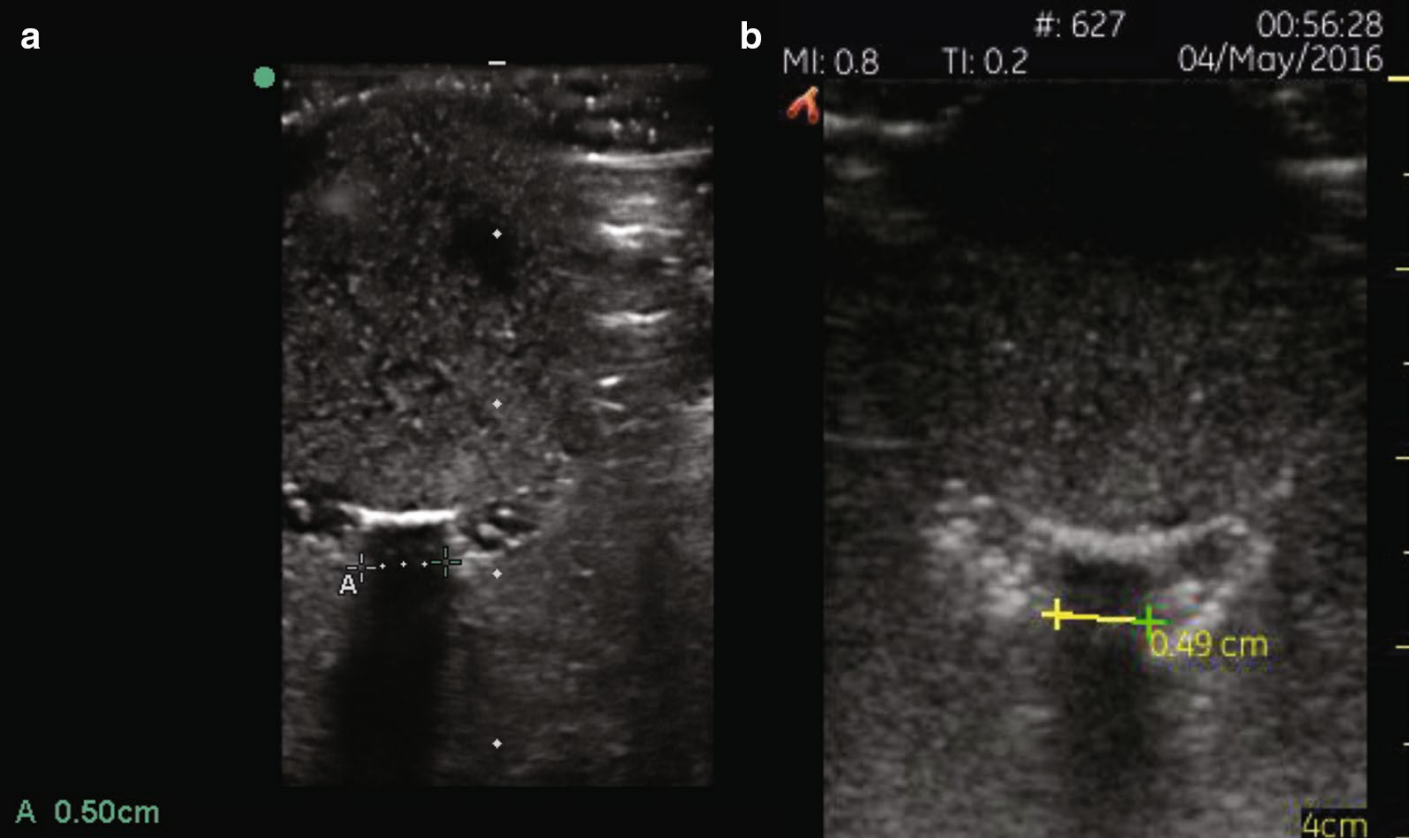
Ultrasound images of ONSD measurements utilizing the SonoSite (**a**) and Vscan (**b**) ultrasound units

We used the standard ultrasound technique for measuring ONSD on our models as previously described [16, 18]. Both a handheld unit (Vscan with Dual Probe utilizing the linear transducer, GE healthcare, Little Chalfont, United Kingdom) and a conventional transducer using a 13–6 MHz linear array (L25x transducer with a SonoSite M-Turbo Ultrasound Machine, SonoSite Inc, Bothell, WA) were used. Sonographers measured each model with both machines (Fig. 1). The models were presented randomly, and the operators were blinded to the color and actual size of each disc. Trials were blocked by machine, where each operator made all of their SonoSite measurements consecutively, before making their Vscan readings.

### Sample size

To compare the level of agreement between the two technologies, we powered our study by assuming a minimum Pearson correlation of 0.6, since less would likely preclude a useful conversion algorithm. Using the Fisher Z transformation with a significance level of 0.05 and a two-sided alternate hypothesis, we calculated that we required 24 measurements to be done with each ultrasound to achieve a 90% power. Therefore, we constructed five different models to be measured in triplicate by two expert operators with both machines for a total of 30 measurements per machine. The disc sizes measured 5.6 (light blue), 5.5 (pink), 6.9 (dark blue), 4.9 (black), and 3.8 (dark green) mm. Disc sizes were chosen to approximate the range of ONSD in patients with normal or elevated ICP found in vivo [6, 8].

### Statistical analysis

Inter-rater reliabilities between operators and between machines were analyzed by intra-class correlation coefficient. Inter-rater reliability between machines (“inter-machine reliability”) was also examined by the Bland–Altman agreement analysis [19]. Finally, both a simple Pearson correlation and quadratic regression were used to compare measurements between machines.

## Results

The ICC between raters for the SonoSite was 0.878, and that for the Vscan was 0.826. The between-machine agreement ICC was 0.752.

**Fig. 2:**
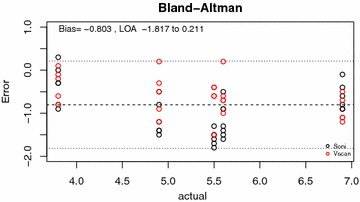
Bland–Altman plot demonstrating the variability of SonoSite (*black*) and Vscan (*red*) measurements over the range of ONSD sizes

**Fig. 3:**
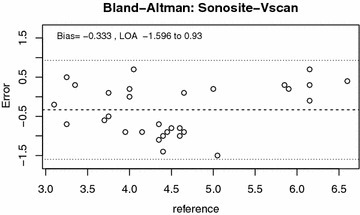
Bland–Altman plot demonstrating the variability of SonoSite–Vscan measurements over the range of ONSD sizes

The Bland–Altman agreement analysis between the SonoSite and the Vscan is shown in Figs. 2 and 3. The Vscan measurements tended to be 0.33 mm higher than the SonoSite on average, with a standard deviation of their difference of 0.64 mm.

**Fig. 4:**
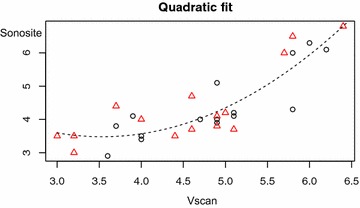
Quadratic regression model comparing VScan to SonoSite measurements

Simple Pearson correlation between machines demonstrated a correlation coefficient of 0.803. Using a quadratic regression, the correlation can be improved and a quadratic equation generated to predict the SonoSite measurement from the Vscan one (Fig. 4). The equation is:

$$y \, = \, 8.54 \, - \, 2.86 \, x \, + \, 0.405 \, x^{2}$$

where *x* is the Vscan measurement and *y* represented the predicted measurement if using the SonoSite machine. After adjusting the Vscan data with this formula, the new mean difference is 0 ± 0.53 mm.

## Discussion

It is currently unknown if pocket ultrasound devices have the resolution to make ONSD measurements as accurately as their larger counterparts. We attempted to assess the accuracy of ONSD measurement in an ultra-portable ultrasound (Vscan) and compare it to a conventional sized portable unit (SonoSite). This was achieved by utilizing our previously validated model, which evaluated intra- and inter-observer variability [16, 18]. We report that the ICC between raters was 0.826 for the Vscan and 0.878 for the SonoSite and the between-machine agreement ICC was 0.752.

Our high inter-rater agreement for both ultrasound devices is equivalent and larger than we have previously reported (ICC = 0.643) using our ONSD model with the same SonoSite device [16]. These improvements are likely due to our exclusive reliance on expert operators for our data this time, as our results are comparable to those found in the previous studies examining inter-rater reliability of experts in small populations in vivo [7, 15, 20, 21]. Although achieving competency in ONSD ultrasonography is not very arduous [22], errors in probe positioning and caliper placement can lead to incorrect measurement with novice operators.

The inter-machine agreement ICC between the SonoSite and the Vscan was 0.752. This is only slightly lower than the ICC between different observers for either machine implying an “excellent” agreement, and that the variation in measurements between the machines was only slightly greater than the variation in measurements between two observers using an identical machine. This suggests that a skilled operator may be able to use either machine to make ONSD measurements with a similar degree of accuracy as two operators using the same machine.

In the Bland–Altman analysis comparing level of agreement between Vscan and SonoSite measurements, there was an even spread across the range of disc sizes suggesting no systematic bias. It did find that the Vscan tended to over-estimate ONSD by a small amount (0.33 ± 0.65 mm). We hypothesize that this may be due to the screen size and resolution of the Vscan as compared to the SonoSite. Since the screen on the Vscan device was smaller than that of the SonoSite, attempts to position, the calipers on the edge of the optic nerve sheath may have lead to an overestimation. Importantly, this strong agreement between ONSD measurements by both devices allows us to create a quadratic conversion formula between the two measures. After adjusting the Vscan results using this formula, the mean difference shrinks to 0 ± 0.53 mm. A conversion formula could potentially be used to translate previously established normal reference ranges from the SonoSite into those more useful for the Vscan. This conversion formula requires further validation in a prospective study.

The advantage of our ONSD model is: it creates a controlled environment where the known disc size was consistent across different measurements and readers. This allows for an isolated comparison of measurement accuracy inherent to each technique and observer. On the surface, comparing the resolution of two ultrasound units to measure a fixed structure may seem like a simple task. The Vscan linear probe has a frequency range of 8–3.4 MHz which is significantly lower than the SonoSite’s 13–6 MHz probe. However, one must consider more than just sheer resolution of the unit, as ultrasound imaging is an operator dependent task that involves optic nerve sheath interpretation, caliper measurement, and ease of software and probe usage. Hence, the primary outcome of agreement between the two measurements is not only important, but also the secondary outcomes of inter-rater and within-subject reliability.

Pocket ultrasound units are not currently approved in Canada or the United States for the measurement of ONSD in human subjects as the existing units do not have the required presets to make these measurements safely. For this reason, we elected to perform this initial pilot study utilizing our ONSD model. The recommended settings for ONSD ultrasound in human subjects requires a thermal index (TI) ≤1 and mechanical index (MI) ≤0.23 [23] which are not achievable with the current Vscan firmware. We, therefore, do not encourage the “off-label” usage of these machines in live human subjects until the appropriate presets are made available.

Despite the importance of our findings, there are limitations to our study. First, all of the data comparing the relative accuracies of the ultrasound devices were obtained in a controlled setting on a simulation model. This was done to limit external variables and limit random error to more precisely study machine and observer effects between devices. However, it is difficult to extrapolate our findings to in vivo as the model does not necessarily account for effects, such as patient variation in anatomy or movement, fluctuations in ICP, and time constraints. Our model may itself introduce some random error of its own, such as imaging the 3D printed disc on an angle causing the disc to produce a larger shadow than usual, or that the shadow generated by the disc is not the same size as the disc itself. We also used a relatively small group of “experts”, as ultrasound evaluation of ONSD is still largely considered experimental in our centre, and there are relatively few experts to recruit. In addition, by having the sonographers both measure using the SonoSite first followed by the Vscan, we may have unintentionally introduced some bias into the measurements, however, looking at the Bland–Altman plot (Fig. 2), the similar variability around the mean for both machines would suggest if present this was likely inconsequential. Finally, our model simulates what is known as the “black stripe method” of ONSD measurement—meaning that the operator measures the edges of the internal diameter of the ONS. However, new quality criteria recently introduced [24] suggest that instead the external hypo-echogenic layers surrounding the optic nerve should be measured. To date, we have been unable to simulate this appearance in our model. This does not invalidate the comparison between ultrasound units are we are still measuring the same structure, but the authors wish to caution the reader that in vivo images may appear different and the ability of the units to clearly differentiate the borders of the ONS may be different in vivo as well.

## Conclusions

This represents the first ONSD study utilizing a pocket-sized, handheld ultrasound device. Despite its small size, lower screen resolution, and significantly lower probe frequency, the ONSD measurements appear accurate and consistent when compared with the conventional portable units. Further study is needed to determine if pocket ultrasound can be accurately used in vivo to measure ONSD.

## Abbreviations

ONSD: Optic nerve sheath diameter
ICP: Intra-cranial pressure
TI: Thermal index
MI: Mechanical index
ICC: Intra-class correlation coefficient
